# Supplementary figures and images for: Activity-based protein profiling reveals both canonical and novel ubiquitin pathway enzymes in Plasmodium
Source: PLoS Pathog. 2025 Apr 18;21(4):e1013032. doi: 10.1371/journal.ppat.1013032 (PMC12007708; doi:10.1371/journal.ppat.1013032)

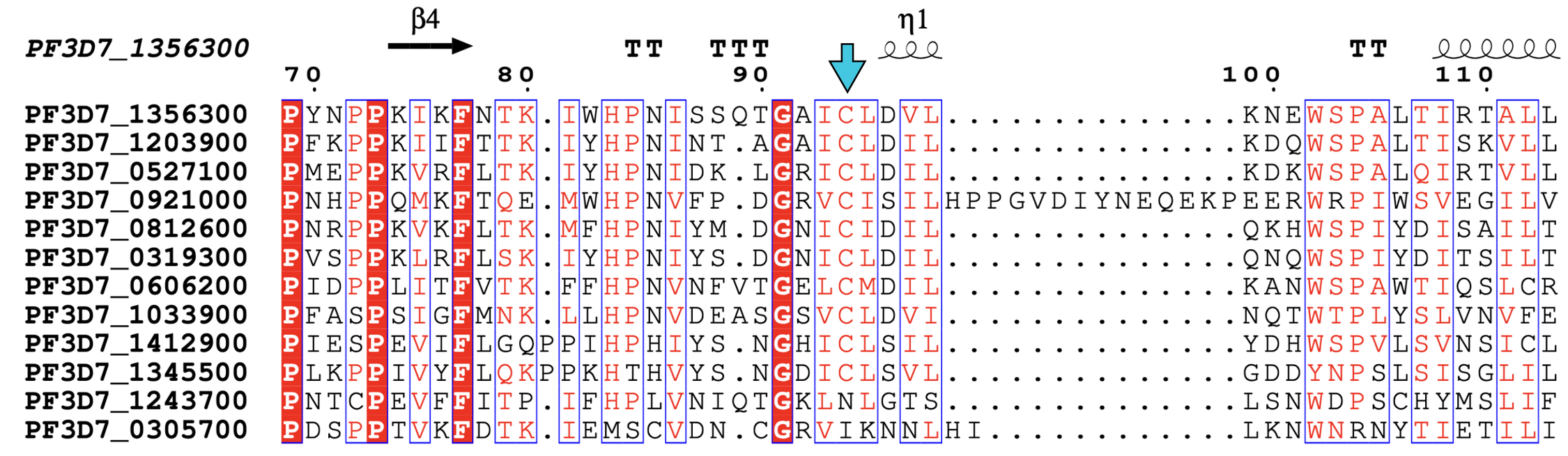

Supplement: S1 Fig — MUSCLE sequence alignment of PfE2 enzymes rendered in ESPript. All annotated PfE2 enzymes contain a conserved cysteine residue (blue arrow), here at numbered residue 94, except two putative UEV enzymes PF3D7_1243700 and PF3D7_0305700. (TIF) [file ppat.1013032.s001.tif]

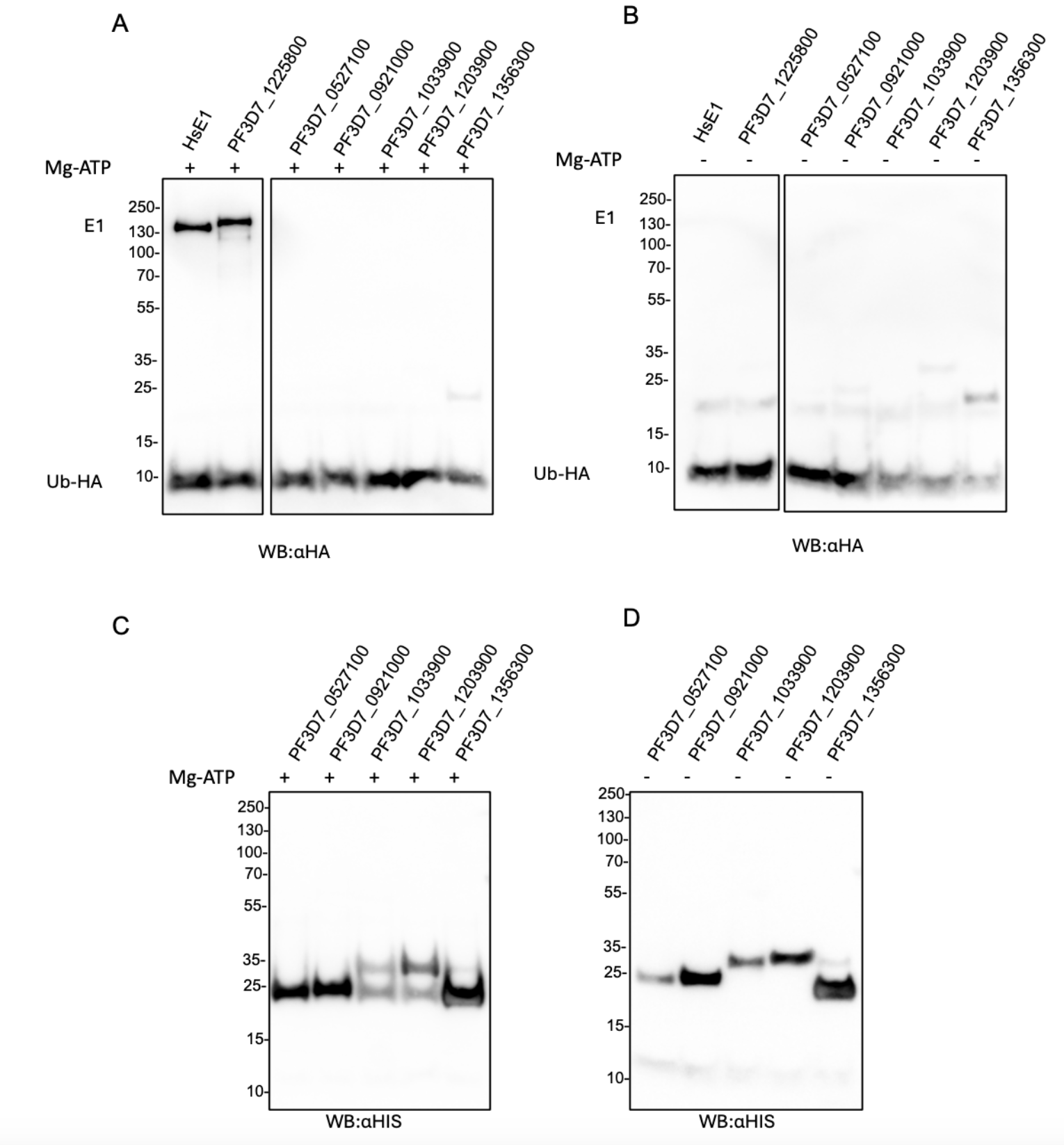

Supplement: S2 Fig — A) Anti-HA blot to demonstrate that E2 activity is ablated in the absence of E1 enzyme for all HIS-E2 enzymes shown in Fig 2. PfE2 enzymes are HIS-tagged and ubiquitin is HA-tagged. B) Additional control showing ablation of E1 activity in absence of Mg-ATP by anti-HA blot to detect ubiquitin. C-D) A repeat of panels A and B but probed with anti-HIS to demonstrate presence of E2 enzymes. (TIF) [file ppat.1013032.s002.tif]

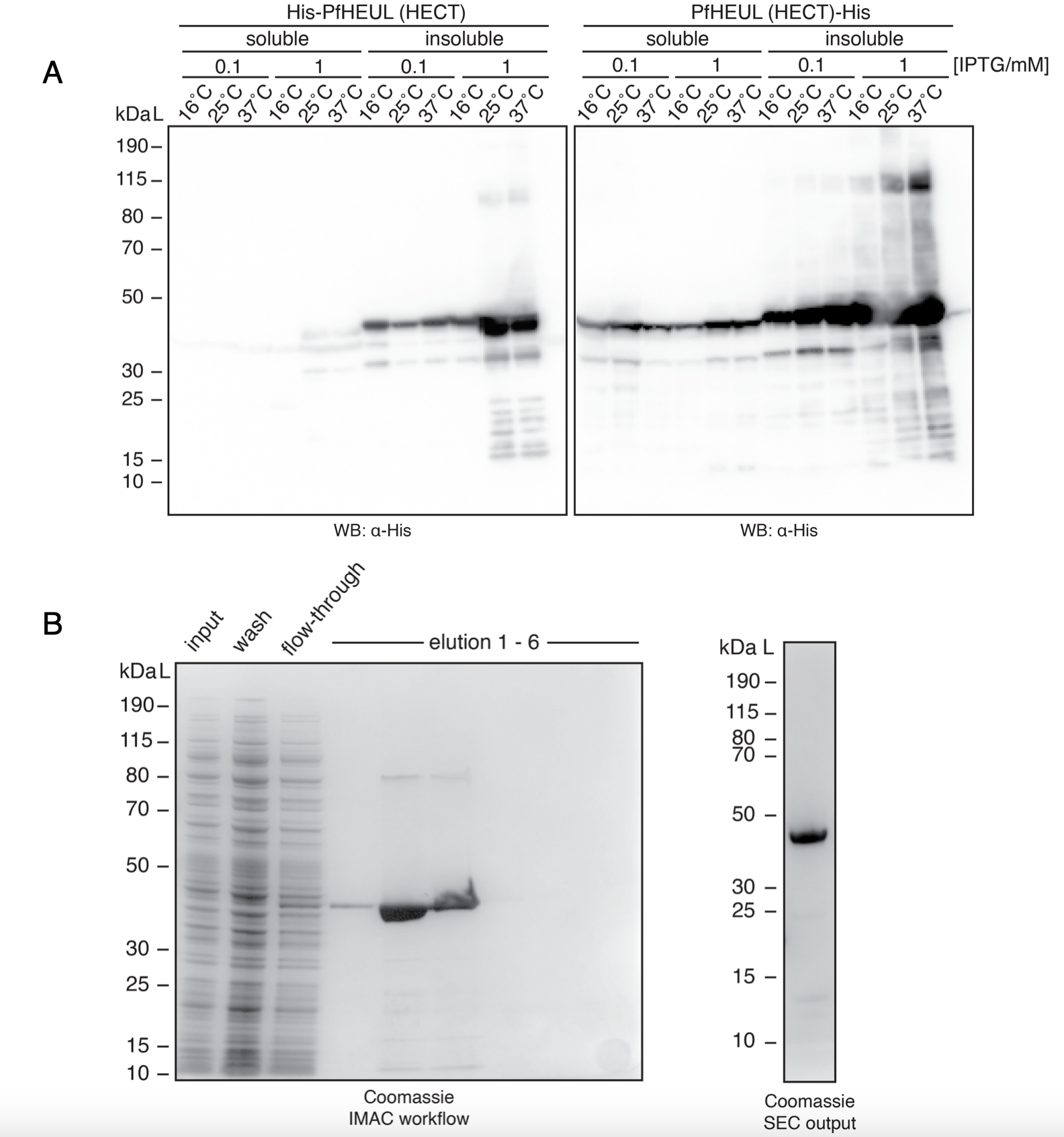

Supplement: S3 Fig — A) Western blot of His-tag PfHEUL HECT (~45 kDa) expression trial under varying incubation temperature and IPTG concentration. Bacteria were chemically lysed and the soluble and insoluble fractions separated by centrifugation prior to resuspension and SDS-PAGE. B) Nickel affinity and desalting workflow assessing input lysate, wash steps, and elution. PfHEUL was eluted maximally by the third elution fraction, and these fractions were pooled and subjected to SEC. This sample demonstrated a single band at the expected molecular weight of PfHEUL. (TIF) [file ppat.1013032.s003.tif]

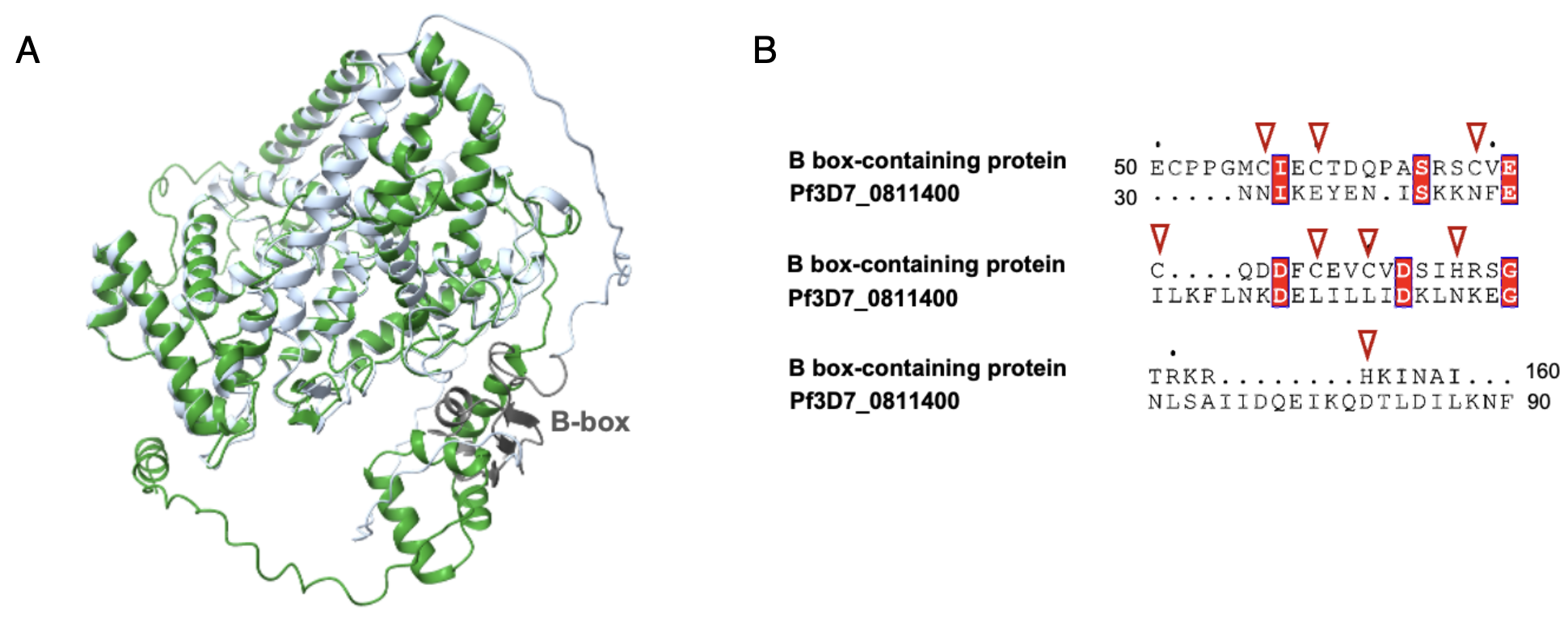

Supplement: S4 Fig — While the C-terminal DUF2009 domain shows a high level of similarity between the two, the B-box domain (highlighted in dark grey) is absent in the N-terminus of Pf3D7_0811400. B. Amino acid sequence alignment of the B-box domain from the B box-type domain-containing protein with the corresponding N-terminal region of Pf3D7_0811400. Cysteines and histidines involved in zinc interaction, indicated by red arrows, are present in the B-box protein but absent in Pf3D7_0811400. (TIF) [file ppat.1013032.s004.tif]

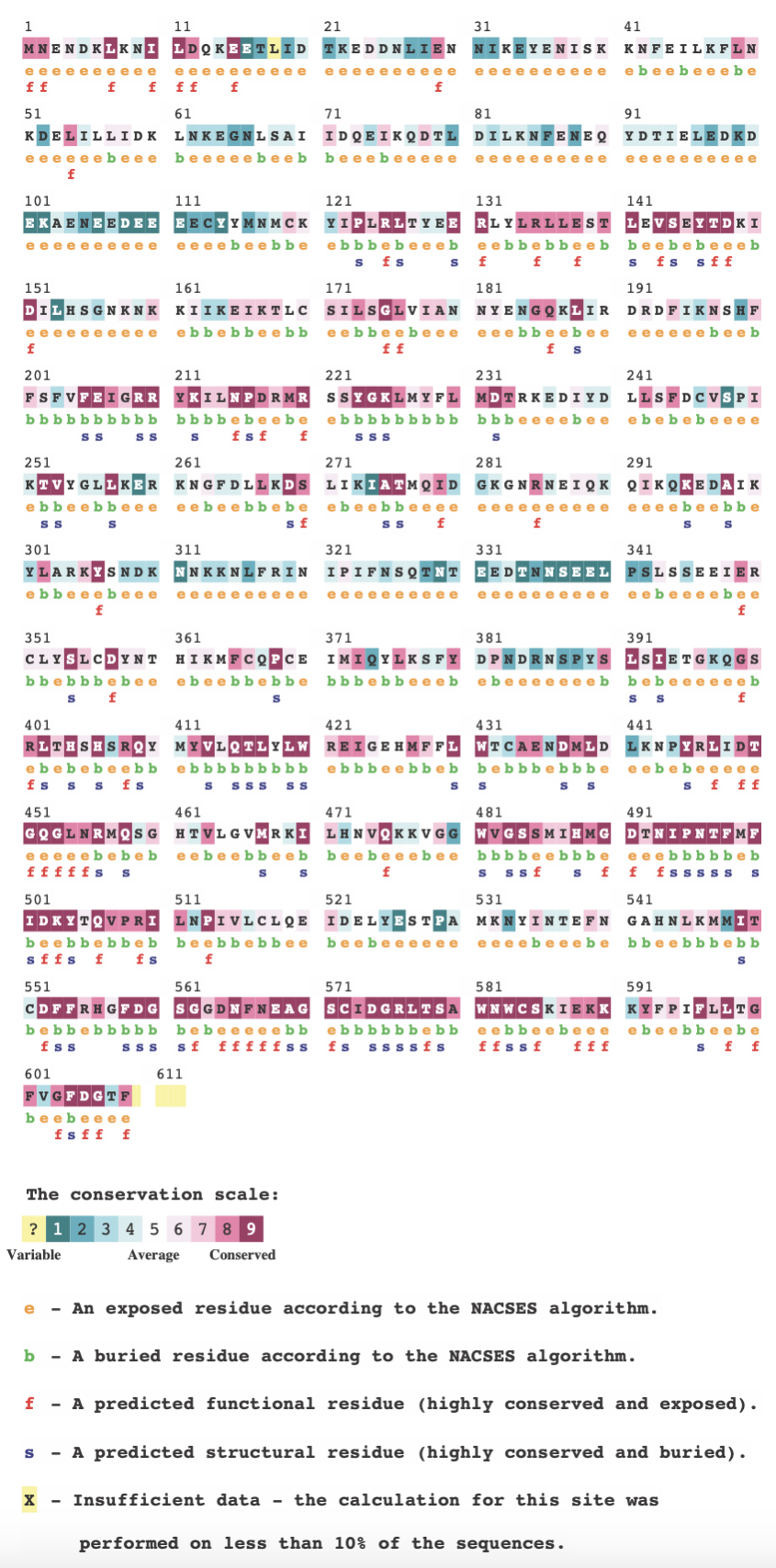

Supplement: S5 Fig — The Consurf web server was used to identify orthologs based on the protein sequence of Pf3D7_0811400. The output was used to colour-code the input PDB structural file (accessed through the AlphaFold web server) based on the computed conservation score for each amino acid residue. Residues are colour-rendered according to the conservation index, with annotations corresponding to the key. (TIF) [file ppat.1013032.s005.tif]

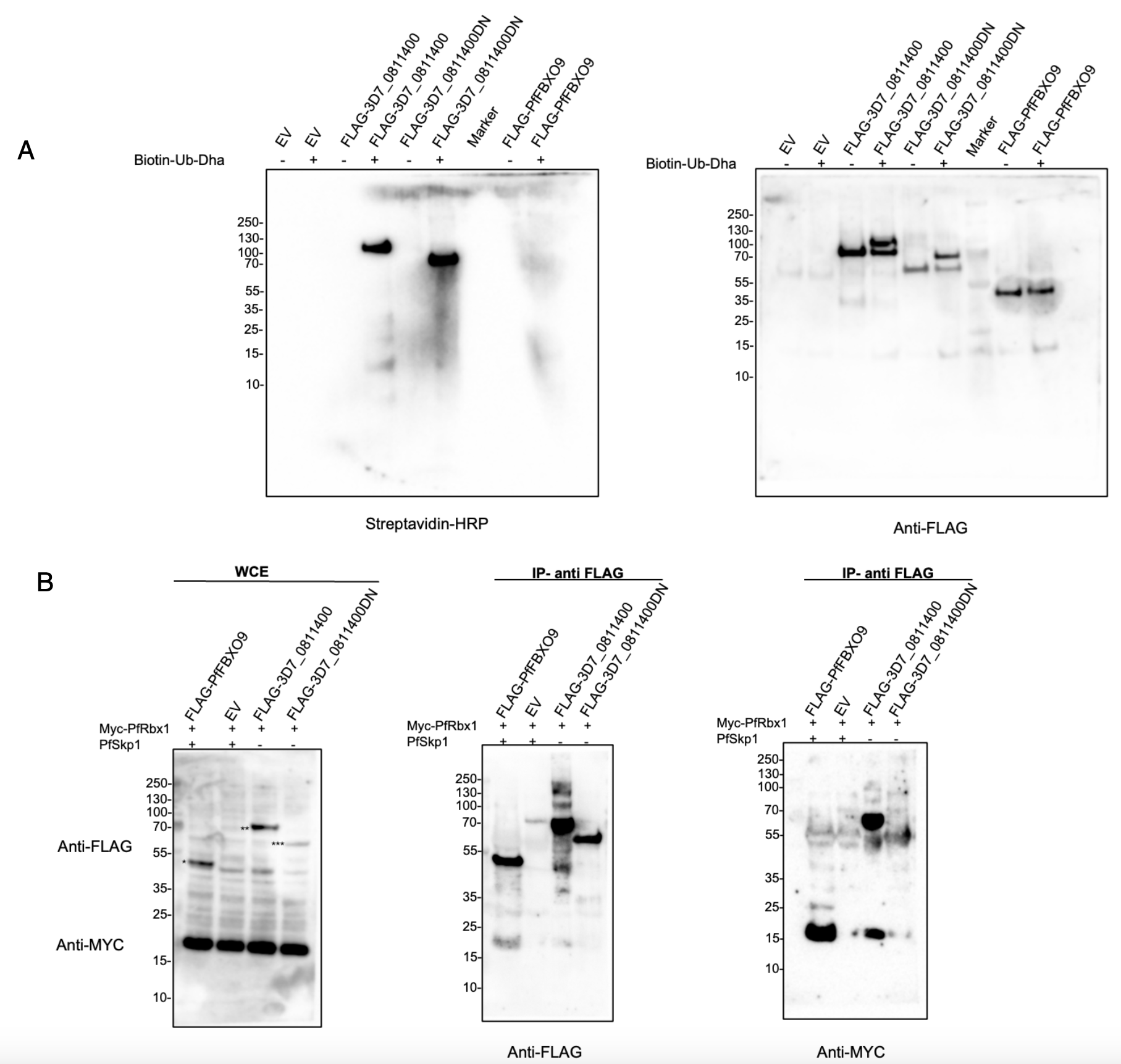

Supplement: S6 Fig — A) Full membranes for Fig 7B streptavidin-HRP (left) and anti-FLAG (right) immunoblots. FLAG-tagged PfFBXO9 served as an additional negative control. B) Full membranes for Fig 7C. The whole cell extract (WCE) membrane was sequentially probed with both anti-FLAG and anti-myc antibodies. The immunopurified proteins were also probed with both antibodies, but on separate membranes (middle image is anti-FLAG and right image is anti-myc). FLAG-tagged Pf3D7_0811400 WT (indicated by**); ΔN N-terminal truncated mutant (indicated by***); FLAG-tagged PfFBXO9 (indicated by*). (TIF) [file ppat.1013032.s006.tif]

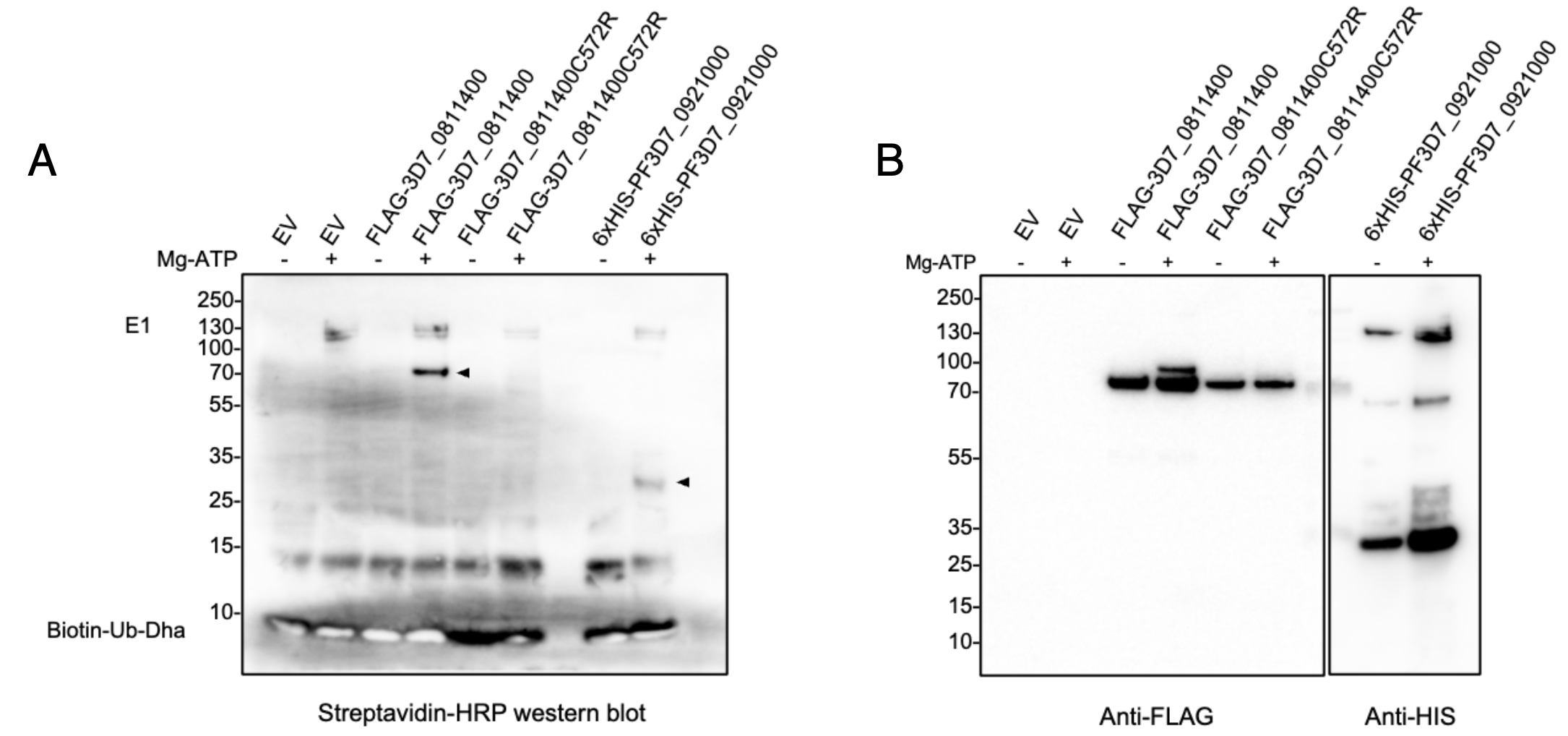

Supplement: S7 Fig — Panels A and B show the full membrane for western blots shown in Fig 8A. Here, a positive control is also included, PF3D7_0921000, a P. falciparum E2 enzyme characterised earlier in this paper. Arrows indicate the signals corresponding to probe interactions with each E2. The FLAG and HIS blots represent the loading of PF3D7_0811400 and PF3D7_0921000, respectively. (TIF) [file ppat.1013032.s007.tif]

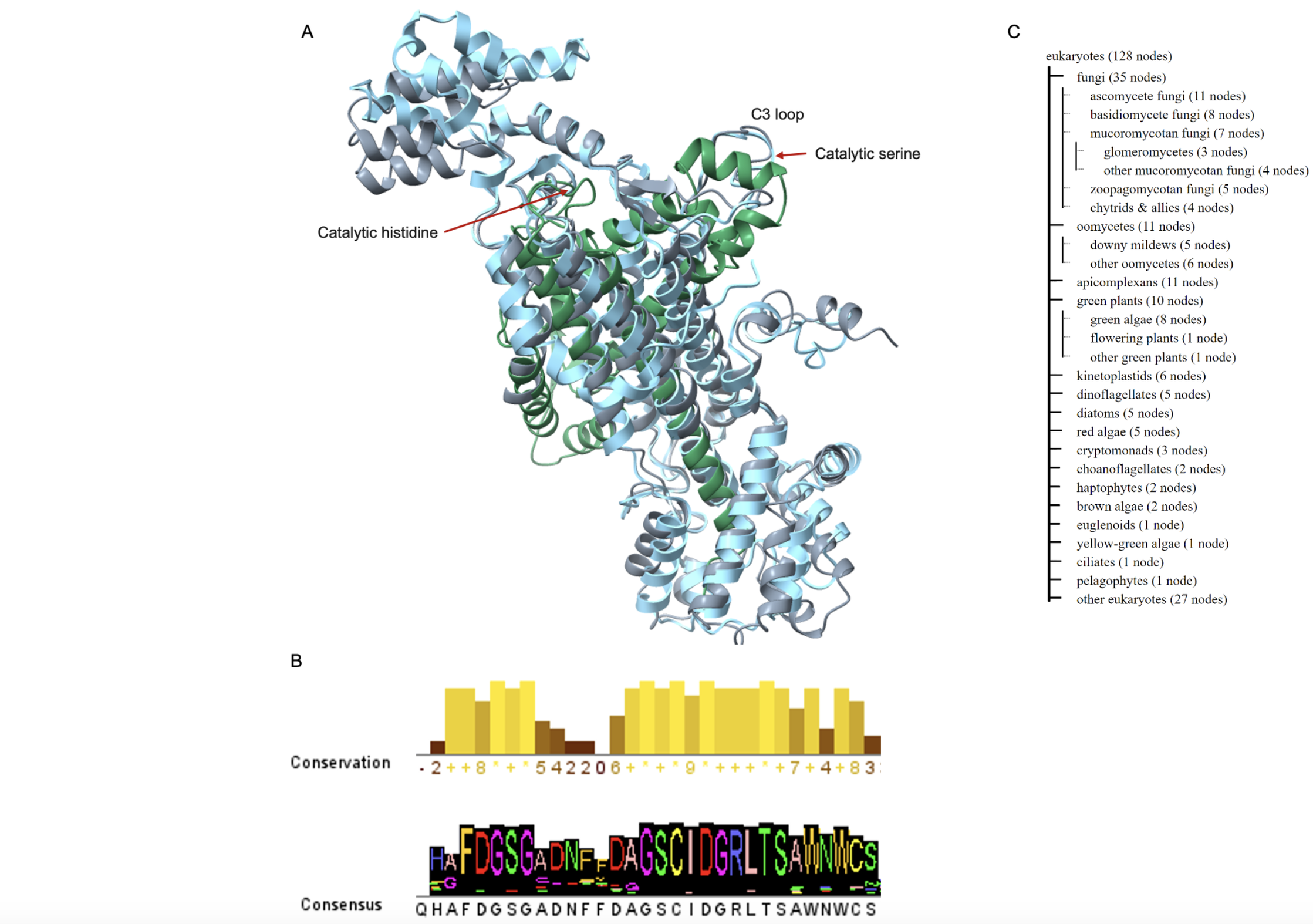

Supplement: S8 Fig — A) 3D structural alignment of the human and Plasmodium ADSL enzymes with the second DUF2009 domain of PF3D7_0811400. While there is high structural similarity between the ADSL enzymes of Plasmodium (gray) and human (blue), PF3D7_0811400 (green) shows only a limited match. Catalytic histidine (Hs H159/ Pf H173) and Catalytic serine (Hs S289/ Pf S298) are conserved in ADSL enzymes but not in DUF2009 domain. Also C3 loop also known as Fumarate lyase, conserved site which is critical for the enzyme activity is absent in PF3D7_0811400. B) Conservation of the catalytic region in 128 eukaryotic proteins with similar structure. Protein sequences were downloaded from UniProt and aligned using MUSCLE, with the alignment visualized in Jalview. Conservation is shown as a histogram, with ‘*’ indicating fully conserved columns (score of 11 using default amino acid property grouping) and ‘+’ marking columns with mutations that preserve all properties (score of 10). C) Classification of the 128 selected proteins based on their corresponding organisms carrying this conserved structure. Data were obtained from the NCBI Taxonomy Browser, and the Common Tree was generated using the NCBI Taxonomy Common Tree tool. (TIF) [file ppat.1013032.s008.tif]
